# Inverse Vulcanized Polymers with Shape Memory, Enhanced Mechanical Properties, and Vitrimer Behavior

**DOI:** 10.1002/anie.202004311

**Published:** 2020-06-04

**Authors:** Peiyao Yan, Wei Zhao, Bowen Zhang, Liang Jiang, Samuel Petcher, Jessica A. Smith, Douglas J. Parker, Andrew I. Cooper, Jingxin Lei, Tom Hasell

**Affiliations:** ^1^ Department of Chemistry University of Liverpool Crown Street Liverpool L69 7ZD UK; ^2^ Leverhulme Research Centre for Functional Materials Design and Materials Innovation Factory University of Liverpool Oxford Street Liverpool L7 3NY UK; ^3^ State Key Laboratory of Polymer Materials Engineering Polymer Research Institute Sichuan University Chengdu 610065 China; ^4^ College of Chemistry and Chemical Engineering Gansu International Scientific and Technological Cooperation Base of Water-Retention Chemical Functional Materials Northwest Normal University Lanzhou 730070 China

**Keywords:** inverse vulcanization, mechanical properties, shape memory, sulfur-polymer, vitrimer

## Abstract

The invention of inverse vulcanization provides great opportunities for generating functional polymers directly from elemental sulfur, an industrial by‐product. However, unsatisfactory mechanical properties have limited the scope for wider applications of these exciting materials. Here, we report an effective synthesis method that significantly improves mechanical properties of sulfur‐polymers and allows control of performance. A linear pre‐polymer containing hydroxyl functional group was produced, which could be stored at room temperature for long periods of time. This pre‐polymer was then further crosslinked by difunctional isocyanate secondary crosslinker. By adjusting the molar ratio of crosslinking functional groups, the tensile strength was controlled, ranging from 0.14±0.01 MPa to 20.17±2.18 MPa, and strain was varied from 11.85±0.88 % to 51.20±5.75 %. Control of hardness, flexibility, solubility and function of the material were also demonstrated. We were able to produce materials with suitable combination of flexibility and strength, with excellent shape memory function. Combined with the unique dynamic property of S−S bonds, these polymer networks have an attractive, vitrimer‐like ability for being reshaped and recycled, despite their crosslinked structures. This new synthesis method could open the door for wider applications of sustainable sulfur‐polymers.

## Graphical abstract

**Functional sulfur**: The synthesis of sulfur‐polymers with significantly improved mechanical properties and controlled performance is reported. In the process a linear pre‐polymer containing hydroxyl functional group is crosslinked by difunctional isocyanate secondary crosslinkers. Control of hardness, flexibility, solubility and function of the material is demonstrated.

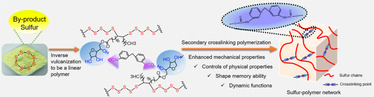

## Introduction

Sulfur, as a by‐product of the purification of crude oil and gas reserves, is widely available and low cost.^1^ The “excess sulfur problem” has drawn the attention of material researchers looking to exploit this underused resource.^2^, ^3^ On the basis of ring‐opening polymerization (ROP) of molecular sulfur rings, researchers have created new materials directly from elemental sulfur to alleviate this problem. To date, multiple routes for producing sulfur‐polymer materials directly from waste sulfur have been proposed, including the reaction of thiols with elemental sulfur,^4^, ^5^ the reaction of element sulfur with *p*‐diiodobenzene,^6^, ^7^ multicomponent polymerizations (MCPs) of sulfur with other molecules,^8^, ^9^ sulfur radical transfer and coupling (SRTC) reaction with benzoxazine compounds,^10^ and inverse vulcanization of sulfur with vinyl groups.^11^, ^12^, ^13^, ^14^, ^15^, ^16^ Among those methods, “inverse vulcanisation”, coined by Pyun et al. in 2013,^11^ has gained much attention for its outstanding benefits: simple, solvent‐free, and high utilization of sulfur. It is notable that inverse vulcanised polymers show various advantageous functions, like mercury capture,^12^, ^13^ self‐healing capability,^17^, ^18^ optical application,^19^, ^20^ electrochemical properties,^21^, ^22^ and antimicrobial properties.^23^

However, poor mechanical properties of these exciting new materials currently limit their wider application and scale of use. Also, there is still little literature on the mechanical properties of inverse vulcanized polymers. According to the available reports,^11^, ^17^, ^18^, ^24^, ^25^, ^26^, ^27^, ^28^, ^29^, ^30^ most materials show a quite low strength compared to conventional polymers. The reported highest stress of this material is 8.69 MPa of copolymer poly(S‐DIB), which means that not much force is required to break the polymers. The change of crosslinkers seem to be the mostly reported method used for improving the related mechanical properties. Either using a new crosslinker or blending two different crosslinkers, the rigidity modulus of sulfur‐polymers could be modified from high to low, but the strength is still always low. This means that polymers can be made either stiff or flexible, but not strong. From a synthetic perspective, constraining ourselves to a one‐step polymerization routes limits the opportunities for realizing multiple performance adjustments.

In a one‐step inverse vulcanisation, it is impossible to control the material properties by adjusting the amount of a crosslinker if the percentage of sulfur is set at a certain desired value. Additionally, once the crosslinking reaction has begun, the crosslinking degree of the polymer is difficult to control. These disadvantages result in few possibilities for significant alteration in physical performances of the polymers through simple replacement of the crosslinker. In this study, we explore a novel synthetic approach that greatly improves the mechanical properties of sulfur‐polymers and could increase opportunities for practical applications. If a linear sulfur‐polymer containing reactive chemical groups could be prepared from inverse vulcanization, then a new network polymer could be obtained through a third monomer crosslinking this pre‐polymer. In this Scheme, an alternative kind of chemical bond can be introduced into the sulfur‐polymer, with the potential to improve its mechanical properties or endow additional functions. Hence, a two‐step polymerization was considered as a method to achieve controllable mechanical properties of sulfur‐polymers by providing the pre‐polymer scope for further modifications. Tsutsumi et al. investigated a similar strategy for the modification of sulfur‐polymers, but they were focused on improving the electrochemical properties of polymers in Li–S batteries application, without characterisation of the physical properties of the materials.^31^

Recently, we showed that a ternary co‐polymer system allows delayed curing to be used,^32^ which could aid practical production, by allowing a liquid pre‐polymer to be transported, stored, and injected into a suitable mould before final setting. From the perspective of practical applications, this one‐step synthesis method was replaced with a two‐step method to generate sulfur‐polymers, anticipating that more promising materials could be obtained. Unlike previous work that has relied solely on crosslinking by reaction of sulfur with C=C bond positions, here we employ a combination of two distinct chemistries: sulfur addition to alkene groups, and reaction of isocyanates with alcohols to form urethane linkages. Hence, we demonstrate that the designed linear polymer formed from sulfur is chemically stable and could be stored at room temperature for long periods of time, and then it could be further modified by the second urethane forming step into a crosslinked polymer. By adjustment of the isothiocyanate crosslink density we demonstrate dramatic increases in the mechanical properties, hardness and solubility of the resultant material. Owing to the unique chemical nature of the produced polymer, some of the samples show a significant shape memory ability. In addition, S−S dynamic bonds give the crosslinked sulfur‐polymer multi dynamic functions, such as reprocess‐ability of polymer network, comparable to vitrimers.^33^, ^34^, ^35^, ^36^, ^37^

## Results and Discussion

**Figure 1:**
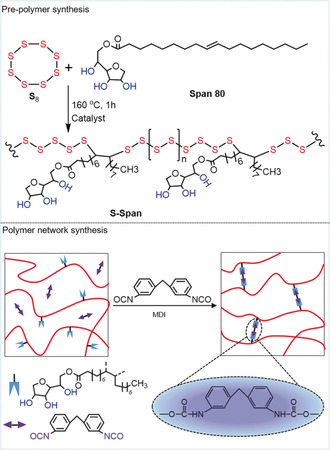
Schematic for the designed inverse vulcanization of pre‐polymer and crosslinked polymer.

Element sulfur exists primarily in the form of an eight‐membered ring (S_8_), which melts on heating and forms polymeric sulfur chains above its floor temperature (159 °C) through a ring opening polymerization process.^9^ However, polymers made purely from sulfur are not stable, and depolymerize back to S_8_, even at room temperature through a back biting mechanism. Based on this principal, a highly crosslinked polymer could be obtained after the addition of small vinylic monomers into liquid sulfur, where they react with the growing sulfur‐polymers, and act to stabilize the material against de‐polymerization. That is the foundation of “inverse vulcanization”. As mentioned above, our aim was to explore more potential properties and wider applications of sulfur‐polymers using two‐step polymerization instead of one‐step method. In this work, span 80 (Span), a trifunctional monomer containing a carbon‐carbon double bond, was selected to stabilize element sulfur to first form a linear pre‐polymer (S‐Span) in the presence of catalyst zinc diethyldithiocarbamate (Zn (DTC)_2_) (Figure 1). As shown in Figure 1, this pre‐polymer with hydroxyl groups on the side chains was further crosslinked by a difunctional monomer diphenylmethane 4, 4′‐diisocyanate (MDI) to generate three‐dimensional sulfur‐polymers (S‐Span‐MDI‐X). Here, X was defined by the molar ratio of ‐OH and ‐NCO, which can be found in Table 1. Detailed illustration and experimental procedures for synthesis of the polymers can be found in the Supporting Information (SI).

**Table 1:** Designed crosslinking degree, thermal and mechanical properties of synthesized polymers.

Sample name	Molar ratio of ‐OH and ‐NCO	Theoretical crosslinking degree [%]	*T*_g_ [°C] (DSC)	*T*_deg,5%_ [°C] (N_2_)	Hardness (HD)	$\sigma_{max}$ (MPa) (average)	Strain_max_ [%] (average)
Poly (S‐Span)	1:0	0	−26.2	190	17.7±0.8	0.14±0.01	35.28±0.98
S‐Span‐MDI‐4	1:0.125	12.5	−20.8	190	34.9±0.6	0.59±0.10	51.20±5.75
S‐Span‐MDI‐3	1:0.25	25	−2.7	186	52.7±1.2	1.88±0.07	42.06±8.25
S‐Span‐MDI‐2	1:0.5	50	28.2	195	63.8±1.3	9.64±1.06	16.08±1.22
S‐Span‐MDI‐1	1:1	100	45.6	210	77.0±1.5	20.17±2.18	11.85±0.88

**Figure 2:**
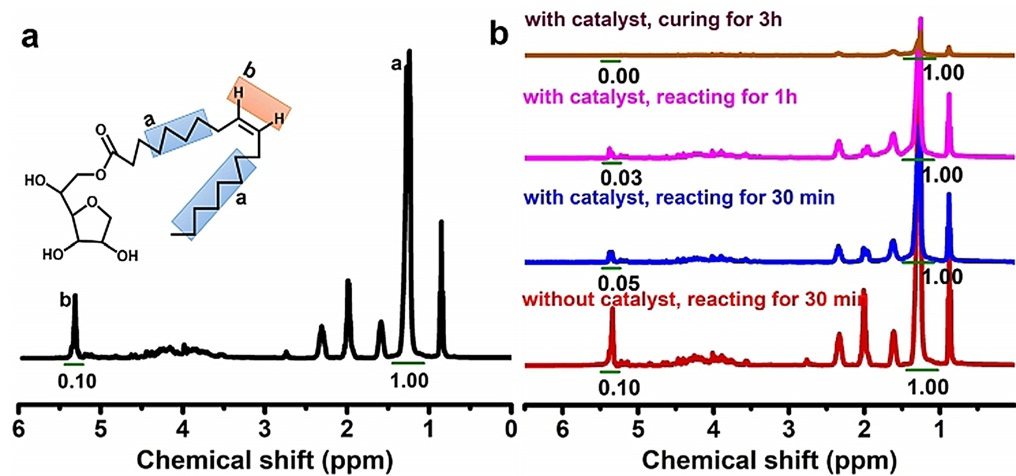
Monitoring the synthesis of pre‐polymer using ^1^H NMR: a) ^1^H NMR spectrum of molecular span 80. b) ^1^H NMR spectra of the mixture during reaction period. From bottom to top, it is responding to without catalyst reacting for 30 min, with catalyst reacting for 30 min and 1 h, and with catalyst curing for 3 h, respectively.

Considering the polymerization activity of two monomers, S_8_ and Span, we need to prove the viability of this polymerization reaction first, and that both monomers are incorporated to form a co‐polymer. Nuclear magnetic resonance spectroscopy (NMR) was performed to monitor this inverse vulcanization reaction of S_8_ with Span (Figure 2). From the reference NMR spectrum of pure Span (Figure 2 a), we can see that the peak (a) at *δ*≈1.3 ppm belongs to ‐CH_2_ groups in ten distinct environments, and the peak (b) at *δ*≈5.3 ppm corresponds to the hydrogens adjacent to the C=C bond. Thanks to unreactive property of the former hydrogen in this reaction, the degree of reaction could be analyzed from comparing the change of integral ratio of these two peaks varying with reacting time. The integral ratio of the two peaks in the spectra (Figure 2 b) varied from 0.1:1, 0.05:1, 0.03:1 to 0:1. This result suggests that C=C bonds were opened as the reaction progressed and finally could be fully consumed in the presence of catalyst Zn (DTC)_2_, a reported effective inverse vulcanization catalyst.^38^ Before curing, when about 80 % of double carbon bonds were consumed (after reacting for 1 h with catalyst), the pre‐polymer S‐Span showed a reversible gel–liquid transformation property and excellent chemical stability at room temperature.

**Figure 3:**
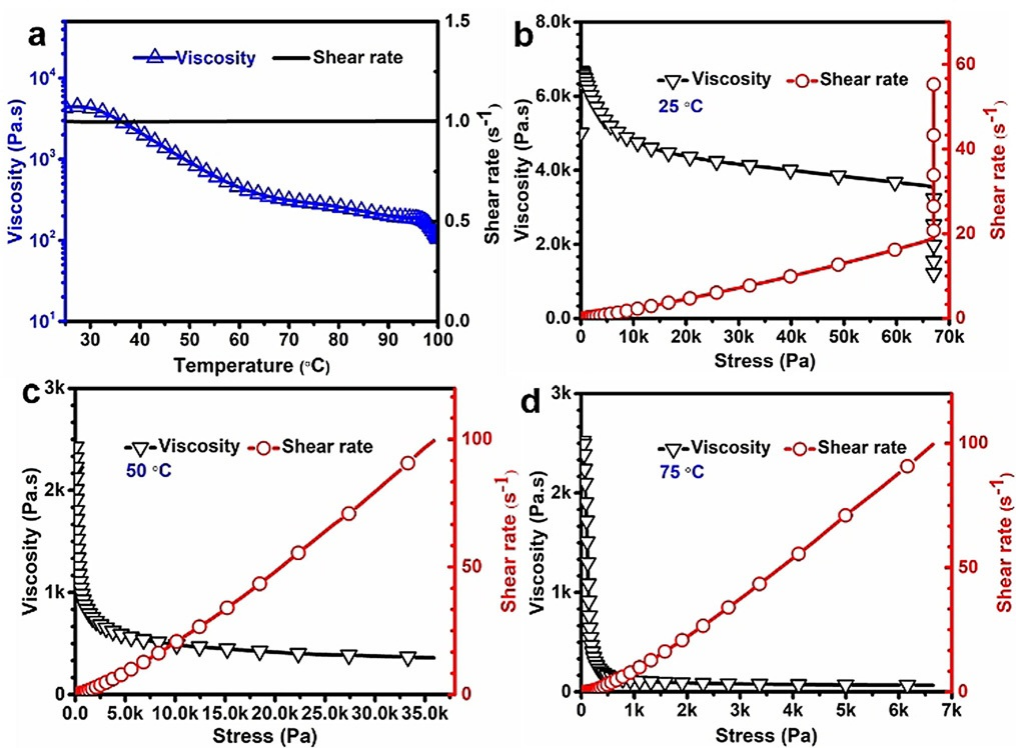
Rheological behavior of pre‐polymer S‐Span: a) Viscosity of pre‐polymer decrease with the increase of temperature at a constant shear rate. b), c) and d) are typical shear‐thinning behavior of pre‐polymer at 25 °C, 50 °C and 75 °C, respectively.

**Figure 4:**
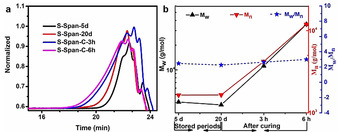
GPC analysis of pre‐polymers. a) Typical molecular weight curves of pre‐polymers from a RI detector. b) A graph demonstrating the weight average molecular weight, number average molecular weight and molecular weight dispersity of pre‐polymers as a function of time at room temperature and subsequent curing time.

The pre‐polymer S‐Span could easily flow with low viscosity at high temperature (above 100 °C), but changed into a gel state with high viscosity and vitrified after cooling down to room temperature (about 20 °C) (Figure S1). Figure 3 a shows that the viscosity of pre‐polymer S‐Span decreases sharply with the increase of temperature under the shear rate control mode of the rheometer. In addition, this pre‐polymer showed a typical shear‐thinning property corresponding to traditional linear polymer behavior, and as a higher temperature was applied less shear force was required to reduce the viscosity of the pre‐polymer (Figures 3 b,c and d). Moreover, this pre‐polymer can be stored at room temperature for long periods, without separation or gelation. From observation using NMR, Differential scanning calorimetry (DSC), Powder X‐ray diffraction (PXRD), Fourier transform infrared spectroscopy (FT‐IR) and Gel permeation chromatography (GPC) (Figures S2, S3, S4, S5 and Figure 4) during storage periods at room temperature, this pre‐polymer exhibited excellent chemical stability, without any structural changes or molecular weight decrease during the observation periods (maximum of 30 days). Therefore, this pre‐polymer could be stored, transported, and be ready for further modification as required.

It was further discovered that before the introduction of the crosslinker MDI into the polymerization system, pre‐polymer S‐Span could be cured into a linear polymer poly (S‐Span) in the solid‐state but lacking in shape persistence ability (Figure S6). As shown by PXRD (Figure S7), no detectable unreacted crystalline S_8_ remained in the polymer after curing for above 3 h. According to FT‐IR (Figure S8), the peak belonging to stretching vibration of C=C−H and C=C in Span at 3080 cm^−1^ and 1600 cm^−1^ disappeared after curing, and there are new peaks at 465 cm^−1^, 550 cm^−1^, and 660 cm^−1^, suggesting that double carbon bonds were fully consumed and new C‐S bonds had been formed. Additionally, as shown in Figure 4, there is an increase in weight average molecular weight (M_w_) with curing time. For example, the M_w_ significantly increased from about 4000 g mol^−1^ before curing, to 11 151 g mol^−1^ after curing for 3 h, and reached at 35 822 g mol^−1^ after curing for 6 h with less change of molecular weight dispersity (M_w_/M_n_ was in the range of 2.5–3) (Figure 3 b). After about 20 h curing, a solid‐state linear polymer poly (S‐Span) with good thermal stability formed (Figure S9) due to the chain entanglement, as confirmed by thermogravimetric analysis (TGA). However, this polymer showed poor shape‐persistence ability at room temperature as its glass transition temperature (*T_g_*) of −26.2 °C is too low to freeze the polymer chains at room temperature, observing from DSC curve (Figure S10), and comparatively low molecular weight allows the solid to be deformed easily.

Motivated by the evidence that long sulfur‐based polymer chains could be formed through S_8_ reaction with Span, and that the pre‐polymer can be stored at room temperature for a long period, the next step was to use a designed method of further modification of the pre‐polymer. Thus, the polymer's performance, like shape retention ability, physical properties, and other potential applications, could be explored and potentially controlled. So, a difunctional isothiocyanate, MDI was selected as the crosslinker to produce polymer networks. The crosslinked polymers S‐Span‐MDI‐X, where X is 1, 2, 3 and 4 responding to the theoretical crosslinking degree of 100 %, 50 %, 25 % and, 12.5 %, respectively, were synthesized and characterized, which are discussed below.

**Figure 5:**
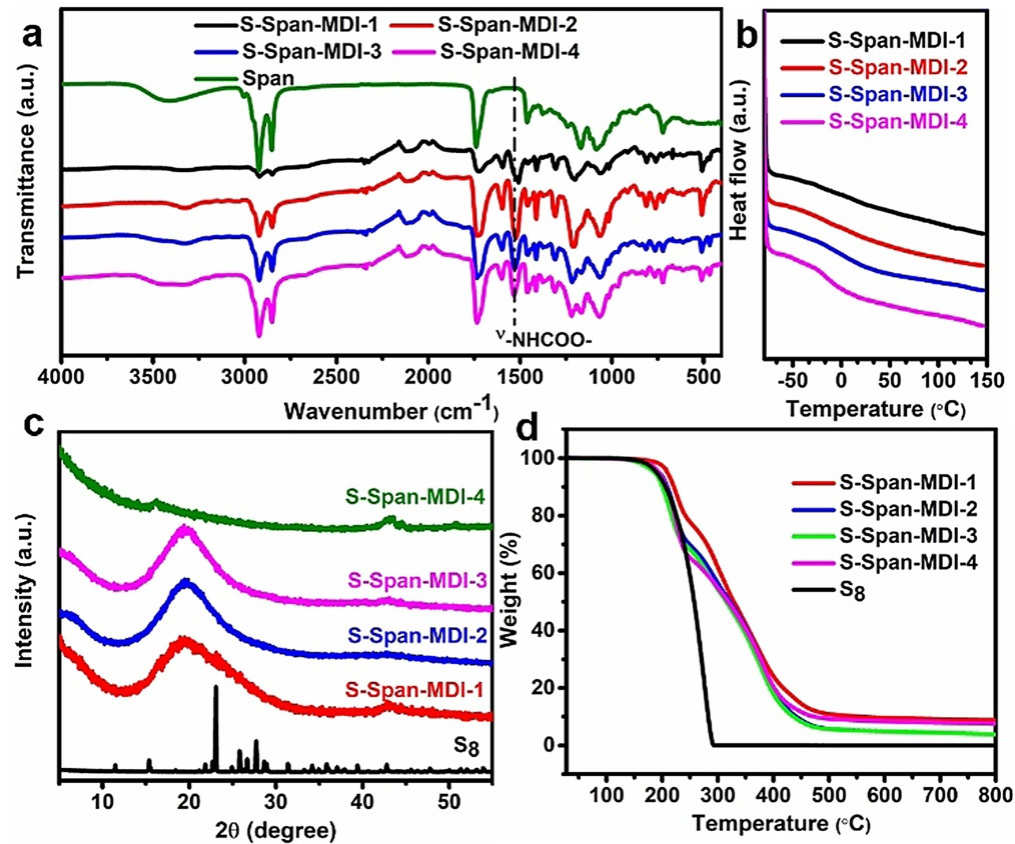
a) FTIR spectra, b) DSC thermograms, c) PXRD patterns and d) TGA thermogram of crosslinked polymers.

Crosslinking density was controlled by adjusting the molar ratio of ‐NCO group, from MDI, and ‐OH group, from Span (Table 1 and SI). Solid ^13^C NMR spectra and FT‐IR were performed to demonstrate that the expected structure of the polymer network had been obtained. Compared to the solution ^13^C NMR spectrum of pure Span in Figure S11, the peak at ≈130 ppm belonging to the carbon in the C=C bond completely disappeared in the solid ^13^C NMR spectrum of poly (S‐Span) (Figure S12). Meanwhile, a new peak at ≈57 ppm was formed, which was attributed to the chemical shift of a C−S bond. That further supports the formation of sulfur‐based polymer from the reaction of Span with S_8_. Afterward, the solid ^13^C NMR spectra of crosslinked polymers were carried out. Taking the solid ^13^C NMR spectrum of crosslinked polymer S‐Span‐MDI‐4 as an example (Figure S13) to analyze, new peaks at ≈57 ppm and ≈154 ppm were attributed to the chemical shifts of C−S bond and ‐NHCOO‐ bond, respectively. Comparable ^13^C NMR results with that of S‐Span‐MDI‐4 were obtained for the other three crosslinked polymers (Figure S14). Moreover, in the FT‐IR spectra shown in Figure 5 a, all the polymers show no unreacted C=C bonds remaining. Also, a new peak was observed at 1530 cm^−1^ corresponding to the amide vibration of ‐NHCOO‐ groups. These results confirm that double carbon bonds were oxidized and consumed, ‐NCO groups reacted with ‐OH groups and carbamate bonds were successfully introduced into the inverse vulcanized sulfur‐polymer. PXRD (Figure 5 c) further proved that no unreacted crystalline sulfur remained in the polymer networks. Therefore, it can be concluded that the sulfur‐polymer networks were successfully synthesized via a two‐step polymerization method, resulting in both S−S and urethane crosslinks.

Solubility experiments were carried out to further characterize the formation of synthesized polymer network structures (SI). The results shown in Figure S15 indicate that as the theoretical crosslinking degree increases, the solubility of the polymers generally decreases. After processing the five polymers by the same procedure, the linear polymer completely dissolved into tetrahydrofuran (THF), dimethylformamide (DMF) and chloroform forming a transparent solution, but the crosslinked polymers showed a tendency for insolubility varying from only partially soluble suspension to an insoluble swelled solid as the degree of theoretical crosslinking increased. Moreover, DSC (Figure 5 b) shows that the *T_g_* of the polymer increased to a higher temperature, but decreased in intensity, with the increase of theoretical crosslinking degree (Table 1). This is explained by the higher degree of crosslinking constraining the polymer chains and requiring higher temperatures for free movement. As the glass transition is a feature of regions of linear polymer chains, increased crosslinking reduces the intensity of this transition. In addition, with the increase of the crosslinking degree, the water contact angle of the polymer also increased. This increase in hydrophobicity is because the concentration of the hydrophilic group, ‐OH group, in the polymers decreases as the crosslinking degree increases. Loss of ‐OH groups is further demonstrated from the decrease of the peak at ≈3400 cm^−1^ in FT‐IR spectra as crosslinking increases (Figure 5 a). TGA in Figure 5 d illustrates that for all four polymer networks, a similar *T*_deg,5 %_ around 200 °C was obtained (Table 1). So, polymer networks with designed crosslinking degrees were successfully obtained.

**Figure 6:**
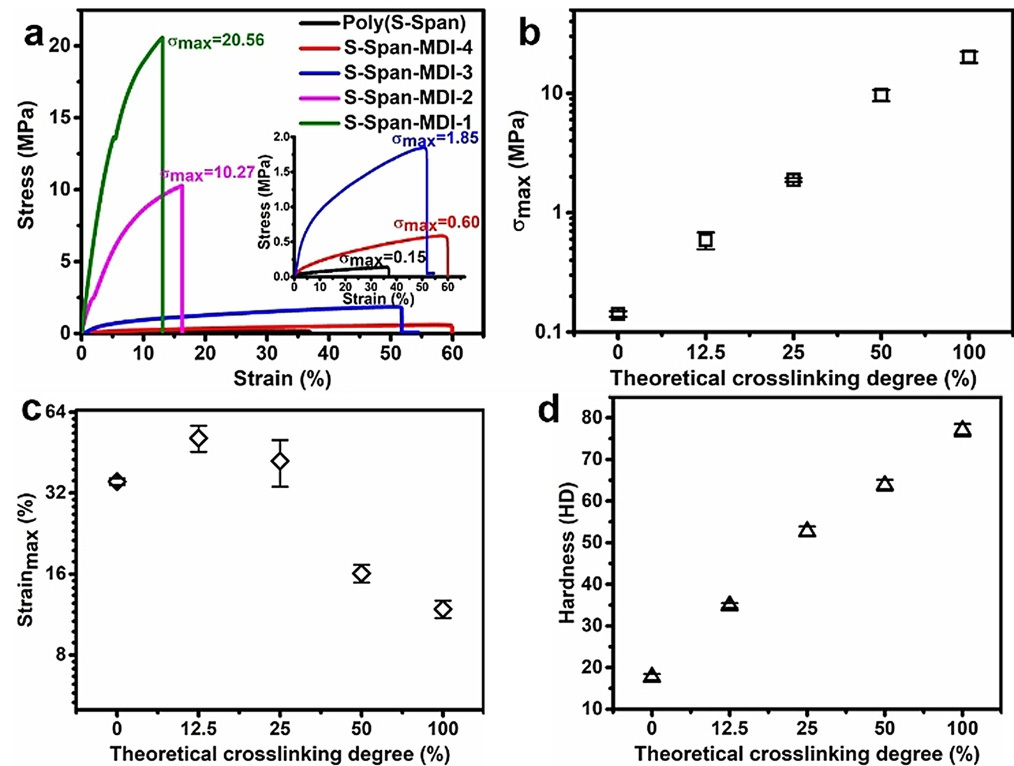
a) The typical strain–stress curves of sulfur‐polymers. b) The stress at break, c) the strain at break and d) hardness of crosslinked polymers plotted against theoretical crosslinking degree.

Generally, chemical crosslinking agents are considered to give enhanced mechanical properties to polymers. Tensile strength measurements were performed on the synthesized polymers, and strong theoretical crosslinking degree dependence was observed. Figure 6 a shows typical strain–stress curves of sulfur‐polymers, indicating that compared to the linear polymer, tensile stress and Young's modulus of crosslinked polymers both have been significantly improved by the chemical crosslinking process. The physical properties of the polymers were controlled, changing from flexible to stiff. The stress and strain at break were analyzed as a function of theoretical crosslinking degree shown in Figures 6 b and c. Stress increased from 0.14±0.01 MPa to a maximum of 20.17±2.18 MPa (a nearly 135‐fold increase). This is attributed to the increase of crosslinking density, as complex three‐dimensional networks with a higher crosslinking degree are not easily destroyed under an external applied force. However, after an increase of breaking strain from linear polymer (35.28±0.98) to slightly crosslinked polymer (51.20±5.75), the breaking strain of crosslinked polymer began a decreasing tendency with the increase of theoretical crosslinking degree from 51.2 % to 11.8 %. It was attributed that crosslinking structure restricts polymer chains from free movement and shape change during tensile deformation. Additionally, due to the difference in structure between the linear polymer and crosslinked variant, different fracture morphology was observed by scanning electron microscopy (SEM). It can be seen in Figure S17 that a cross‐section of the linear polymer is noticeably rougher than that of crosslinked polymers. Meanwhile, the changing chemical structure endows different hardness to the polymers (Table 1). As shown in Figure 6 d, the hardness of the polymers increases from 17.7±0.8 HD of linear polymer to 77.0±1.5 HD of fully crosslinked polymer. It can be seen that the polymers show an obvious trend from soft to hard, as shown in photographs in Figure S18.

S−S bonds have been demonstrated to show a thermally induced dynamic exchange reaction, and dynamic S−S bonds have been widely reported to be applied in vitrimers to achieve recyclability of traditional thermoset polymers.^39^, ^40^, ^41^ Additionally, an inverse vulcanized sulfur‐polymer was recently reported as s functional crosslinker for epoxy thermosets to endow the epoxy material potential self‐healing ability due to the particular property of dynamic S−S bonds.^42^ Hence, it is plausible that our sulfur‐polymers, the backbone of which is formed of sulfur chains, should possess dynamic properties associated with disulfide vitrimers. The dynamic property of crosslinked polymers induced by S−S bonds was demonstrated by using dynamic mechanical analysis (DMA). Fully crosslinked polymers are not usually able to be reprocessed or recycled, as this would require the irreversible breakage of C−C bonds and degrade the network. Whereas when dynamic chemical bonds are introduced into a polymer, it becomes reprocessable due to the potential for topological rearrangement caused by dynamic covalent chemistry. So, here fully crosslinked polymer S‐Span‐MDI‐1 was selected to characterize the stress relaxation behavior at varying temperatures. If polymer S‐Span‐MDI‐1 could be proved to show an obvious stress relaxation property, the other three crosslinked polymers with a lower crosslinking degree definitely should have the same ability.

**Figure 7:**
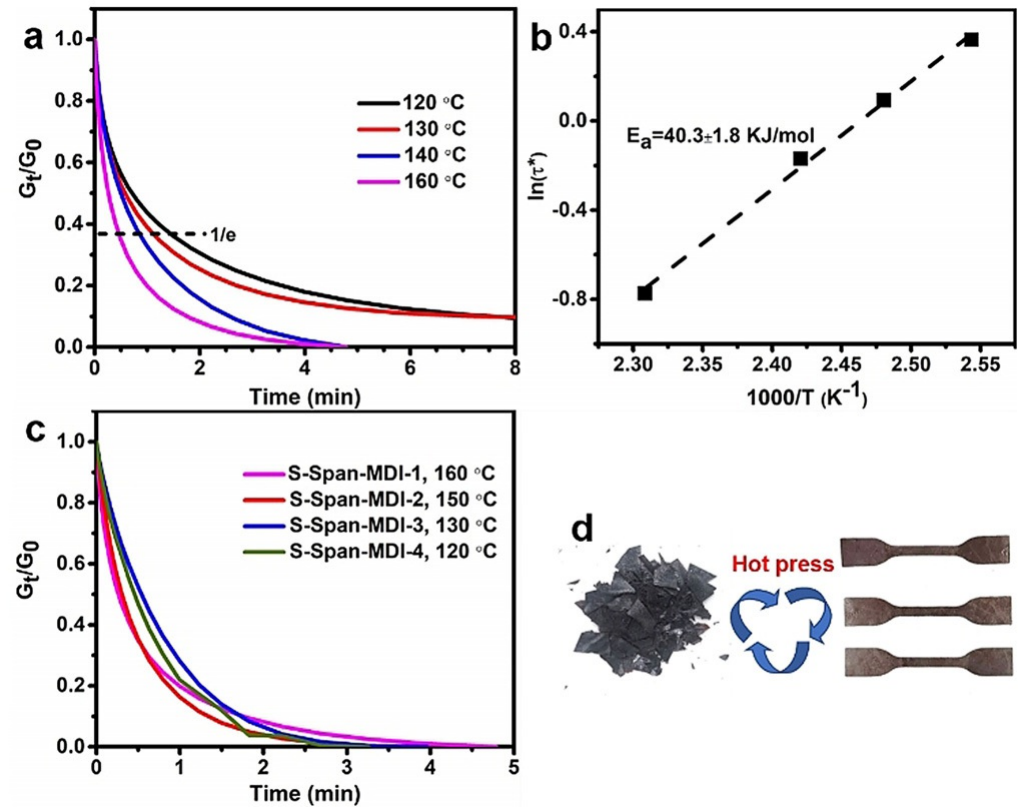
a) Stress relaxation behavior of fully crosslinked polymer S‐Span‐MDI‐1 varying with temperature investigated by DMA. b) Arrhenius plot of polymer S‐Span‐MDI‐1 with a linear correlation. c) Stress relaxation behavior of crosslinked polymers at pre‐determined temperature. d) A diagram demonstrating a typical recycling experiment of polymer S‐Span‐MDI‐1.

The stress relaxation characterization of polymer S‐Span‐MDI‐1 was carried out at 120 °C, 130 °C, 140 °C, and 160 °C under control of strain at 1 %. Figure 7 a shows that stress was able to relax to zero at a high temperature within 5 min. It was indicated that quicker relaxation happened at a higher temperature. When the results were plotted in an Arrhenius plot, a linear correlation was obtained and the activation energy of 40.3 kJ mol^−1^ was calculated from the slope [SI Eq. (1)]. The other three crosslinked polymers show similar dynamic properties to each other, by appropriately controlling the temperature (Figure 7 c). With the decrease of crosslinking degree, the temperature required for similar stress relaxation time with S‐Span‐MDI‐1 at 160 °C decreases. These results prompted further investigation into the recycling of crosslinked sulfur‐polymers. Reprocessing experiments were carried out by cutting up the sulfur‐polymers, before reforming them using a hot press (Figure 7 d).

**Figure 8:**
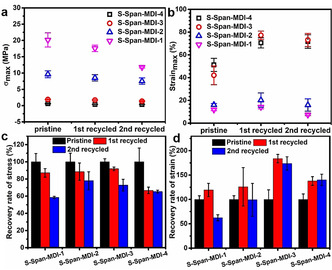
a) The breaking stress and b) the breaking strain of crosslinked polymers before recycling and after two repeats of recycling. c) The recovery rate of maximum stress and d) the recovery rate of maximum strain of crosslinked polymers after each time they were reprocessed.

To be clear, the original samples were also formed by use of a hot press after oven molding, but are marked as pristine in Figure 8, as they had not been intentionally recycled. The exact values of breaking stress and strain of the crosslinked polymers before and after reprocessing are shown in Figures 8 a and b. The stress decreased after every recycling step, with the highest recovery after the first cycle being 91.3 % recovery for S‐Span‐MDI‐3 and highest after the second cycle 78.0 % recovery of S‐Span‐MDI‐2 (Figure 8 c). From the FT‐IR spectra (Figure S19) and PXRD curves (Figure S2) of pristine and reprocessed samples, no structure change is observed after reprocessing experiments. To explain the incomplete recovery of stress, we would attribute some minor chemical degradation resulting from thermal stress exerted via the hot press. However, except for S‐Span‐MDI‐1 after the second cycle, which showed a decrease in strain, all crosslinked polymers show a significant strain increase after every time they are recycled (Figure 8 d). That was considered to be caused by homogenization of the sulfur chain lengths during rearrangement in the thermal reprocessing process, and/or some breakage of the polyurethane crosslinks. Both of these hypotheses are consistent with the slight decrease of *T*_g_ after reprocessing evidenced from DSC (Figure S21). Under the same testing temperature, the polymer with lower *T*_g_ can be further deformed without break as the polymer chains are more mobile under stretching force. So, it is clear that the dynamic S−S bonds endow recyclability to the crosslinked polymers whilst simultaneously preserving the crosslinked structure.

The shape memory function of traditional polymers has been studied for many years, including principles and applications.^43^, ^44^, ^45^ However, the shape memory of inverse vulcanized sulfur‐polymers has not been reported to date. The limit of weak or stiff mechanical properties of such sulfur‐polymers may be considered as the main challenge for not achieving shape memory function. We have discussed above that sulfur‐polymers with controlled physical properties can be obtained. Among them, crosslinked polymers S‐Span‐MDI‐1 and S‐Span‐MDI‐2 show an excellent temporary shape maintenance effect at room temperature. As polymer S‐Span‐MDI‐2 has a suitable *T*_g_ of 28.2 °C and is more flexible than S‐Span‐MDI‐1 at room temperature, it was selected as an example to discuss that property. While studying the shape memory property of polymer S‐Span‐MDI‐X, two related sulfur‐polymer materials, made through different methods, were reported.^5^, ^7^ Traditionally, chemical crosslinking leads to thermoset shape memory polymers with robust shape memory but low recycling ability. However, the introduction of dynamic bonds provides the crosslinked shape memory polymers with the ability to also be recycled. Despite the reversible shape‐memory transitions, the polymer could also obtain a new shape caused by topological rearrangement from the dynamic reaction of the reversible S−S bonds. Here, the distinct elastic shape memory and plastic permanent reshape property of a crosslinked sulfur‐polymer was investigated.

**Figure 9:**
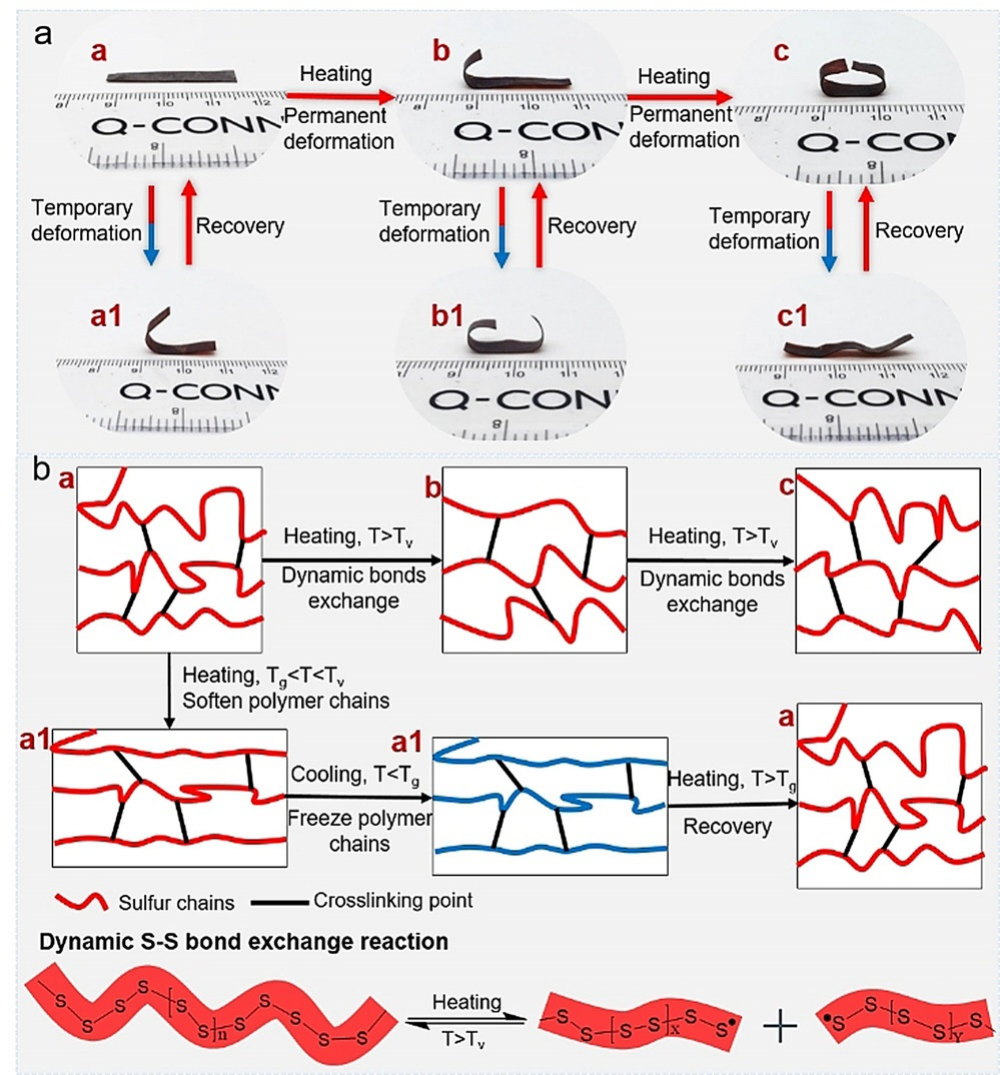
a) Example of reversible shape memory performance and the cumulative plasticity reprogramming process of crosslinked polymer S‐Span‐MDI‐2. b) Schematic for explaining how the dynamic property of the polymer is caused by polymer structure changes.

As shown in Figure 9 a, the shape (a), a rectangular film, was able to be reshaped, by heating above the *T*_g_ and then cooling, into a temporary shape (a1) that can recover when reheated. The recovered shape (a) can be further deformed into a permanent shape (b) by heating to higher temperature (solid–solid transition temperature (*T*_v_)) allowing dynamic S−S bond exchange. This second permanent shape (b), is then still capable of further temporary shape deformation and recovery, as before (b1). After that, the recovered shape (b) can still be further reshaped to a third distinct form (c) following again by a reversible shape memory behavior (c1). Two videos of this process are available in the supporting information, corresponding to the reversible shape memory behaviors between shape (a) and shape (a1) and between shape (c) and shape (c1). The fundamental principle of this distinct elasticity and plasticity property is the perfect combination of phase change and topological rearrangement induced by temperature. As illustrated by Figure 9 b, the film can be heated to its glass transition temperature first and then reshaped by an external force. This temporary shape can be maintained by freezing its polymer chains after cooling down and can recover to the original shape by heating again. The dynamic S−S bond exchange reaction, which can be triggered by heating up to the *T*_v_, results in the topological rearrangement of the polymer network. So, a permanent shape of the film can be obtained through the thermally induced dynamic exchange reaction. And this behavior can be repeatedly carried out without loss of the temporary shape memory effects of the pristine material, suggesting that a cumulative reshaping of this sulfur‐polymer network can be obtained as discussed above.

## Conclusion

In summary, we designed and synthesized pre‐polymer and polymer networks, with variable degrees of crosslinking, directly using by‐product elemental sulfur through a two‐step polymerization method. The pre‐polymer is chemically stable, is able to be stored at room temperature for long periods of time, and is ready for further modification when needed. By adjusting the degree of crosslinking during the second, chemically distinct, reaction step, the physical properties such as glass transition temperature, solvent resistance, hardness, contact angle, and mechanical properties of the polymers were effectively controlled. The polymers show a clear tendency, varying from weak and soft to strong but hard with the increase of crosslinking degree. In addition, the polymer network with a suitable degree of crosslinking shows an excellent shape memory effect. The unique dynamic property of S−S bonds provides the synthesized sulfur‐polymer networks with reprocessing and plasticity reshaping abilities. We have realized enhancing the strength of sulfur‐polymers (e.g. >20 MPa tensile strength, an increase of ≈135 times), but combining such high strength with high flexibility for sulfur‐polymers is still challenging. There is still great potential for a wider range of crosslinking degrees and crosslinking agents to be exploring as a way of further tuning the properties of sulfur‐polymers to meet various practical needs. We believe that the basic principles of this work can be expanded into a range of applications and other research areas.

## Conflict of interest

The authors declare no conflict of interest.

## Supporting information

As a service to our authors and readers, this journal provides supporting information supplied by the authors. Such materials are peer reviewed and may be re‐organized for online delivery, but are not copy‐edited or typeset. Technical support issues arising from supporting information (other than missing files) should be addressed to the authors.

Supplementary

Supplementary

Supplementary
