# Supplementary material for: Inverse Vulcanized Polymers with Shape Memory, Enhanced Mechanical Properties, and Vitrimer Behavior
Source: Angew Chem Int Ed Engl. 2020 Jun 4;59(32):13371–8. doi: 10.1002/anie.202004311 (PMC7497146; doi:10.1002/anie.202004311)
Supplement: Supplementary file 1 — Supplementary [file ANIE-59-13371-s001.pdf]

Angewandte Chemie
Eine Zeitschrift der Gesellschaft Deutscher Chemiker

Supporting Information

Inverse Vulcanized Polymers with Shape Memory, Enhanced Mechanical Properties, and Vitrimer Behavior

*Peiyao Yan, Wei Zhao, Bowen Zhang, Liang Jiang, Samuel Petcher, Jessica A. Smith, Douglas J. Parker, Andrew I. Cooper, Jingxin Lei, and Tom Hasell**

anie_202004311_sm_miscellaneous_information.pdf
anie_202004311_sm_shape_a1.mp4
anie_202004311_sm_shape_c1.mp4

Table of contents

Experimental section

1. Materials

2. Characterization

3. Experimental procedure

Results and discussion

References

Experimental section

Materials

Sulfur (S_8, ≥99.5 %), Span 80 (Span) (428.62 g/mol), Diphenylmethane 4,4'-diisocyanate (MDI) (250.25 g/mol), Zn diethyldithiocarbamate (Zn $(DTC)_2$), Chloroform-d ($CDCl_3$), Dimethylformamide (DMF), Chloroform, Acetone, Acetonitrile, Toluene, Tetrahydrofuran (THF), Methanol and Hexane were purchased from Sigma Aldrich and used as received without further purification.

Characterization

1. *Nuclear magnetic resonance spectroscopy (NMR)*: Solution NMR was recorded in deuterated chloroform using a Bruker Advance DRX (400 MHz) spectrometer. $CDCl_3$ was used as solvent.

2. *Solid-state ^{13}C NMR measurement*: Carbon-13 magic-angle spinning measurements were carried out at 100.63 MHz using a Bruker Advance III HD spectrometer and 4 mm (rotor o.d.) probe. Spectra were acquired at a spin rate of 10 kHz. Cross-polarisation (CP) spectra were recorded with TOSS spinning sideband suppression, 1 ms contact time and with a recycle delay of 2 s. Carbon spectral referencing is relative to neat tetramethyl silane, carried out by setting the high frequency signal from an external sample of adamantane to 38.5 ppm. Samples were cut into ~1×1 mm squares.

3. *Fourier transform infrared spectroscopy (FT-IR)*: FT-IR was carried out using Vertex 70 Fourier Transform Infrared Spectrometer, between 400 cm^{-1} to 4000 cm^{-1}.

4. *Differential scanning calorimetry (DSC)*: DSC was carried out using Q2000 DSC (TA instruments). The method was a heat/cool/heat for three cycles at a heating/cooling rate of 10 °C/min and with ranging from -80 ºC to 150 ºC. The second heating curves were recorded and analysed.

5. *Thermogravimetric analysis (TGA)*: The thermal stability of sulfur-polymers was conducted using a TA Instruments Q500 under nitrogen from room temperature to 800 °C at a heating rate of 20 °C min^{-1}.

6. *Powder X-ray Diffraction (PXRD)*: Crystal fraction of polymer was detected using a PANalytical X'Pert PRO diffractometer with Cu-$K_{\alpha1+2}$ radiation, operating in transmission geometry.

7. *Rheological characterization*: Rheological behavior of pre-polymer was evaluated by using the TA instrument Rheometer Ares G2 with 25 mm parallel plates (PPS). The viscosity of pre-polymer was tested at temperature ranging from 20 °C to 100 ºC with 5 °C increments and under shear rate of $1s^{-1}$. Shear-shinning property of pre-polymer was measured under initial shear rate 0.01 to final 100 s^{-1} at three different temperatures (25 °C, 50 °C and 75 °C).

8. *Gel permeation chromatography (GPC)*: The molecular weight of pre-polymer was measured by using Viscotek TDA302 GPC (THF). All the concentrations of samples were prepared as 5 mg/ml.

9. *Stress relaxation*: Stress relaxation of the crosslinked polymers was tested by using TA Instruments Q800 Dynamic Mechanical Analysis (DMA). Samples were prepared as rectangular shapes with same dimensions. Controlling the tensile strain at 1%, the stress of samples was allowed to relax to zero at different temperatures. Under designed temperature, stress relaxation behaviour (G_t/G_0)-time (t) dependence was recorded. Stress relaxation activation energy, E_a, was calculated by fitting the Arrhenius equation[1]:

$$\ln\tau^{*}(T) = ln\tau_0 + E_a/RT \qquad \text{Equation (1)}$$

Where τ^* is the relaxation time when the value of G_t/G_0 reaches e^{-1}; *R* is the gas constant 8.314 J K^{-1} mol; Temperature is thermodynamic temperature. By fitting τ^* with 1000/*T* in software, E_a will be calculated out.

10. *Tensile test*: Tensile stress-strain curves of polymers were carried out using a universal testing Machine (SHIMADZU EZTest) with crosshead rate of 5 mm/min. The testing temperature was fixed at 22 °C using air conditioner. Dog-bone samples were made from polymer films responding to ISO 527-2/5A. The experimental procedure of making polymer films is illustrated in the next section. The average values with error bar of all mechanical data were obtained after 3~5 times tests for every sample.

11. *Hardness*: Hardness of polymers were evaluated using D type Digital Shore Durometer. Same thickness of 1 mm was applied to all the polymers, and above 7 times tests of hardness for every polymer were conducted out in order to obtain a reliable average value.

12. *Drop shape*: Contact angle of polymers with water was conducted out using Kruss DSA100 Expert Drop Shape Analyser. Testing temperature was 24 °C and fitting method used is Ellipse.

13. *Scanning electron microscope (SEM)*: The cross-sections after tensile breaking were observed on Hitachi SEM S4800.

Experimental procedure

1. *Synthesis of pre-polymer S-Span*

The total mass of raw materials was set to 10 g, the weight percent of sulfur and Span were 50 wt. % and 50 wt. %, respectively. 5 g (0.0195 mol) of sulfur was added to a 40 mL glass reaction vail equipped with a magnetic stir bar and was heated on a hot plate to 160 °C. After the orange sulfur liquid was formed, 5 g (0.0117 mol) of Span and catalyst Zn $(DTC)_2$ (100 mg) were added at stirring speed of 900 rpm. After reacting for 1 h, the pre-polymer was obtained after the mixture cooling down naturally to room temperature. This pre-polymer was named as S-Span.

2. *Synthesis of polymer poly (S-Span)*

The pre-polymer, which was directly cured into solid polymer without adding any crosslinker, was named as poly (S-Span). The experimental procedure for synthesizing poly (S-Span) is described as below. The total mass of raw materials was set to 10 g, the weight percent of sulfur and Span were 50 wt. % and 50 wt. %, respectively. 5 g (0.0195 mol) of sulfur was added to a 40 mL glass reaction vail equipped with a magnetic stir bar and was heated on a hot plate to 160 °C. After the orange sulfur liquid was formed, 5 g (0.0117 mol) of Span and catalyst Zn $(DTC)_2$ (100 mg) were added at stirring speed of 900 rpm. After reacting for 1h 20

min, the whole mixture was poured into a silicone mould followed by 2 minutes cooling down and ~20 h curing at 130 °C in the oven.

3. *Synthesis of crosslinked polymers S-Span-MDI-X*

The crosslinked polymers with different theoretical crosslinking degree were designed and polymerized. Those polymers were named as S-Span-MDI-1, S-Span-MDI-2, S-Span-MDI-3 and S-Span-MDI-4, responding to the molar ratio of –OH and –NCO of 1:1, 1:0.5, 1:0.25 and 1:0.125, respectively. Before the crosslinker MDI was added, pre-polymer was synthesized first as followed description. 5 g (0.0195 mol) of sulfur was added to a 40 mL glass reaction vail equipped with a magnetic stir bar and was heated on a hot plate to 160 °C. After the orange sulfur liquid was formed, 5 g (0.0117 mol) of Span and catalyst Zn $(DTC)_2$ (100 mg) were added at stirring speed of 900 rpm. After reacting for 1h 20 min, predetermined stoichiometric amount of MDI was added. After stirring for another 3 min, the whole mixture was poured into a silicone mould following by the curing for ~20 h at 130 °C in the oven.

4. *Monitoring the pre-polymer S-Span during stored periods*

One pre-polymer S-Span was prepared first and cooled down to room temperature. This sample was stored at room temperature for one month, and ^{1}H NMR, DSC, PXRD and FTIR were used to monitor the structure change during stored periods. The data was recorded after storing for 1 day, 3 days, 5 days, 10 days, 20 days and 30 days, and the corresponding sample was named as S-Span-1d, S-Span-3d, S-Span-5d, S-Span-10d, S-Span-20d and S-Span-30d, respectively.

5. *Film making and reprocessing experiments using hot press*

The polymers were hot pressed into uniform films, and also were reprocessed, using a Hydraulic Lamination Hot Press purchased from Zhengzhou CY Scientific Instrument CO., LTD. Polymer films with average thickness of 1.00 mm were obtained through hot pressing inside a square metal mould with prescribed thickness. For original samples, the polymers were directly hot pressed after curing in the oven without any further processing. For recycling experiments, all polymers were cut into pieces then were hot pressed into new films. In principle, higher crosslinking density of the polymer requires higher temperature and longer pressing time to get a complete film. According to the results concluded from DMA characterization, the reprocessing procedure was optimised as follows: Generally, S-Span-MDI-1 was hot pressed under 20 MPa at 160 °C for 25 min, S-Span-MDI-2 was hot pressed under 20 MPa at 150 °C for 15 min, S-Span-MDI-3 was hot pressed under 20 MPa at 130 °C for 10 min, S-Span-MDI-4 was hot pressed under 20 MPa at 120 °C for 5 min, and poly (S-Span) was hot pressed under 20 MPa at 110 °C for 5 min. Then, after cooling down to room temperature and unloading the mould, polymer films with the same thickness were obtained.

6. *Solubility evaluation*

THF, Chloroform, DMF and Toluene were chosen as solvents to research the solubility of the sulfur polymers. The concentration of polymer was prepared above 20 mg/mL in 10 ml of the solvent. All the samples were stirred by stirrer bar for 5 h at 50 °C with 400 rpm. After stirring, the soluble polymer could form a transparent liquid in a good solvent, with colour change, but

insoluble polymers could only be swelled by solvents without significant colour change. The changes for five polymers before and after stirring in four solvents, regardless of the solution or solid state of polymers, were recorded by photographs (Results shown in Figure S15).

7. *Shape memory and permanent reshaping experiments*

The polymer S-Span-MDI-2 was cut into a rectangular film and then was performed three cumulative reshaped deformations with three reversible temporary shape memory behaviour cycles. The film was temporarily reshaped at 80 °C for seconds and maintained its shape at room temperature (about 20 °C), and then that temporary shape got a reversible recovery at 80 °C during seconds. For permanent reshaping, the film was reconfigured at 140 °C for 15 min and maintained its shape at room temperature. As the topological rearrangement of the film results in a permanent reshape, the new shape does not recover to the original shape.

Results and discussion

Synthesis and characterization of pre-polymer S-Span

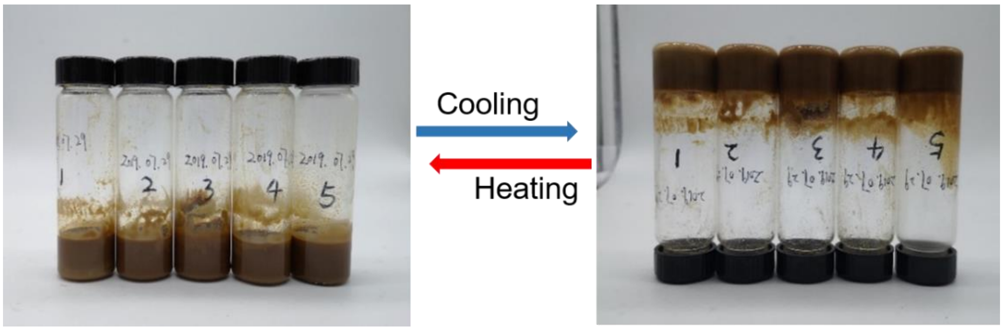

Figure S1. Photographs of the Gel-liquid transformation property of pre-polymer S-Span. Before curing, the pre-polymer was in a liquid state and could flow. It became solid after cooling down, and then could change back to liquid again after heating up. Therefore, it was theorized that the pre-polymer can be stored at room temperature for long periods of time without change of structure, and then can be heated up into liquid for further modifications.

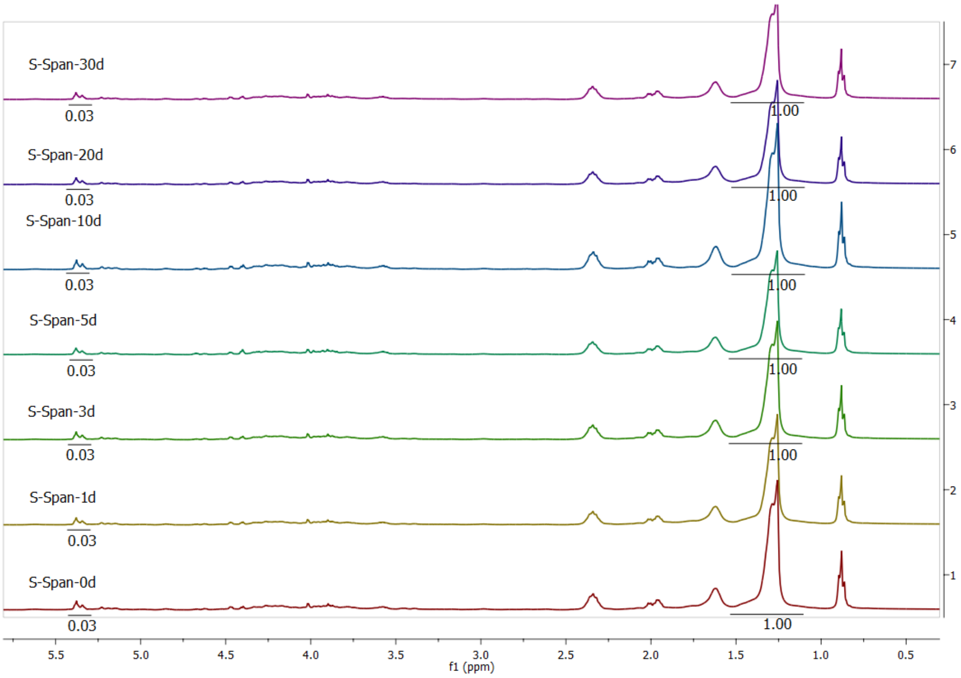

Figure S2. ^{1}H NMR was performed to monitor the structure change of pre-polymer during stored periods. From bottom to top, the NMR spectrum corresponds to pre-polymer stored for 0 day, 1 day, 3 days, 5 days, 10 days, 20 days and 30 days, respectively. It is clear from the spectra that there was no structure change happened during the stored periods, as the integral ration between two kinds of hydrogen stayed at the same value (0.03:1.00).

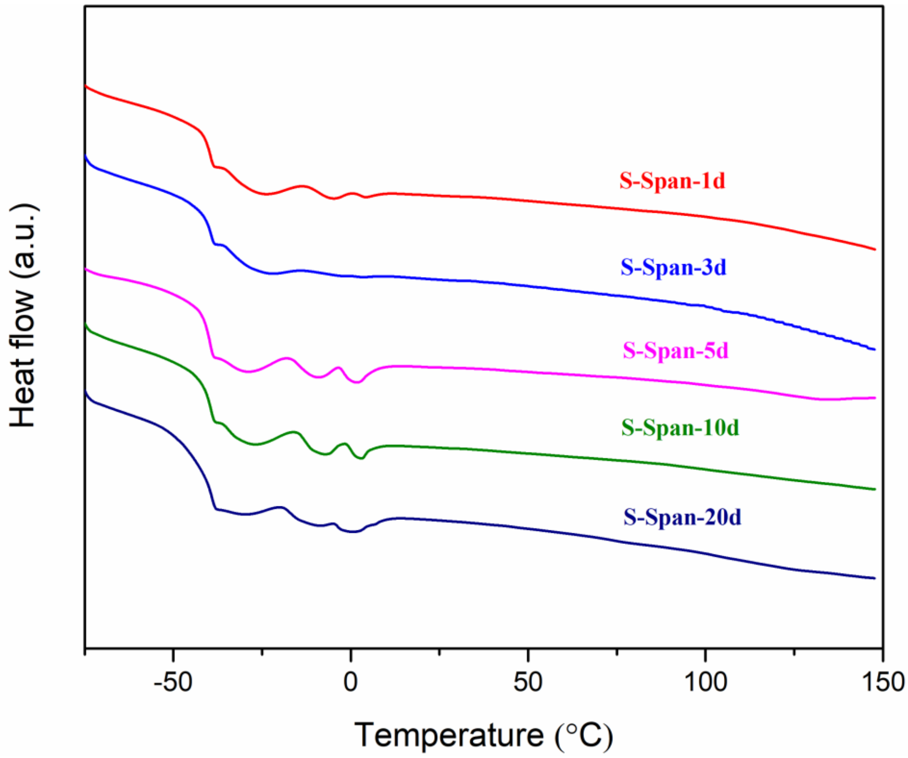

Figure S3. DSC was performed to monitor the structure change of pre-polymer during stored periods. As shown in the figure, when the temperature was increased, unreacted monomers continued reacting induced by heating, but there is no change of thermal property of pre-polymer during this period.

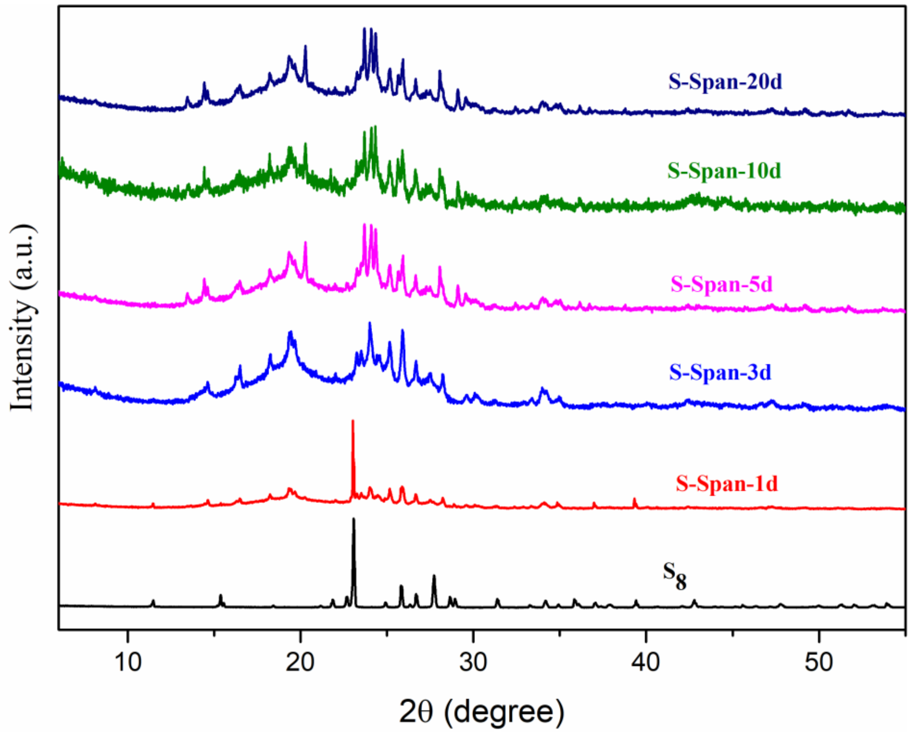

Figure S4. PXRD was performed to monitor the structure change of pre-polymer during stored periods. As shown in figure, there were unreactive crystalline S_8 remaining suspended in the pre-polymer, and there is no increase of crystal signals from pre-polymer during this stored period, suggesting no additional sulfur precipitated out during this period.

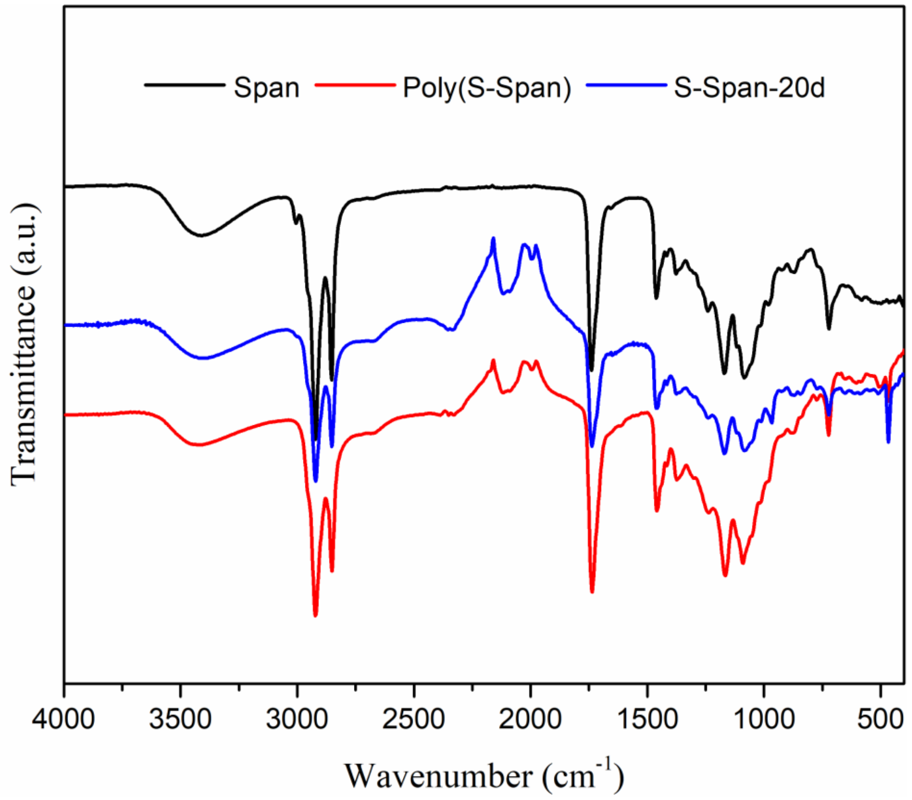

Figure S5. FT-IR was performed to monitor the structure change of pre-polymer during stored periods. As shown in figure, no obvious peaks belonging to double carbon bonds (at 3080 cm^{-1} and 1600 cm-1) could be found in pre-polymer, and there is no new peak formed and no old peak disappeared, indicating that there was no structure change during this stored period.

Synthesis and characterization of linear polymer poly (S-Span)

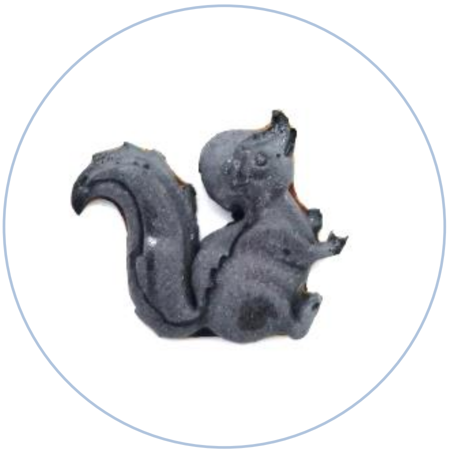

Figure S6. Moulded sample from linear polymer poly (S-Span). This sample was easily deformed under external force at room temperature and was sticky at temperature above 20 °C resulting from its low glass transition temperature, and relatively low molecular weight.

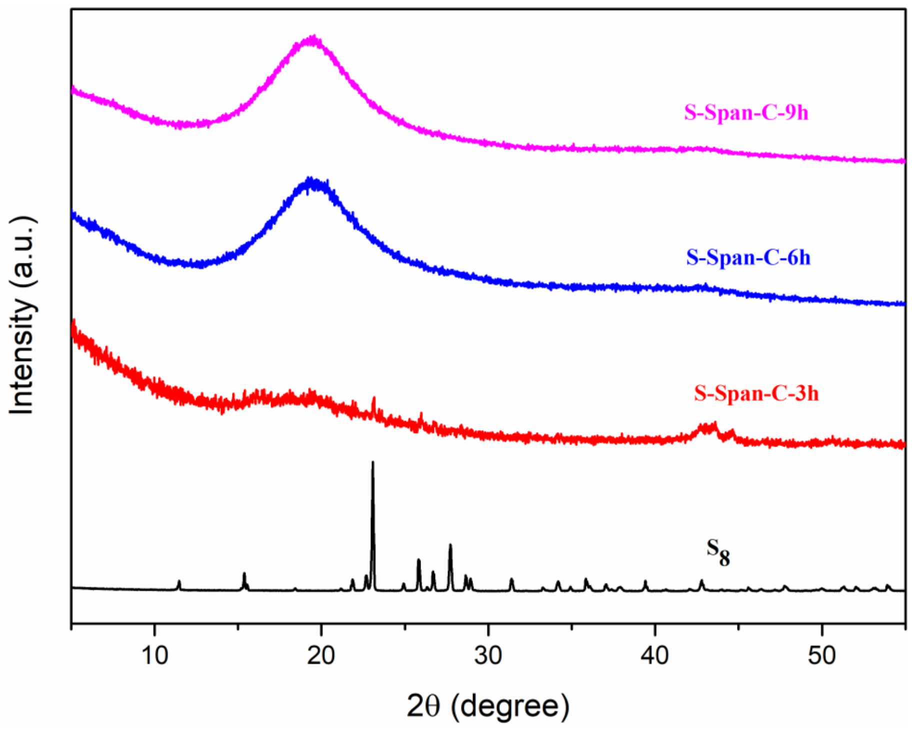

Figure S7. Offset PXRD patterns of pre-polymer cured for different periods of time. The samples were named as S-Span-C-x, where x refer to curing for 3 h, 6 h and 9 h. As the figure shown, after curing for 3 h, there were still some crystal signals apparent from polymer, but after curing for above 6 h, there was no crystalline S_8 remaining in the polymer. Therefore, the linear polymer poly (S-Span) in the solid state was obtained after curing for ~20 h.

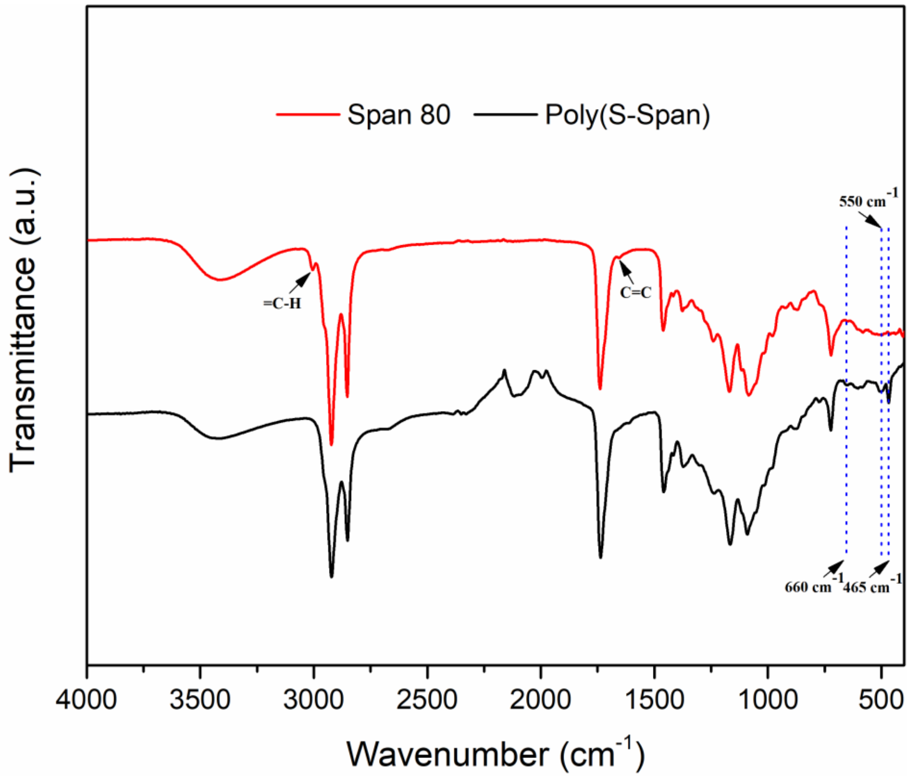

Figure S8. FT-IR trace of linear polymer formed from sulfur. The peak belonging to stretching vibration of C=C-H and C=C in Span at 3080 cm^{-1} and 1600 cm^{-1} totally disappeared after curing, and there are two new peaks at 465 cm^{-1}, 550 cm^{-1} and 660 cm^{-1} appeared, suggesting that double carbon bonds were fully consumed and new bond C-S had been formed.

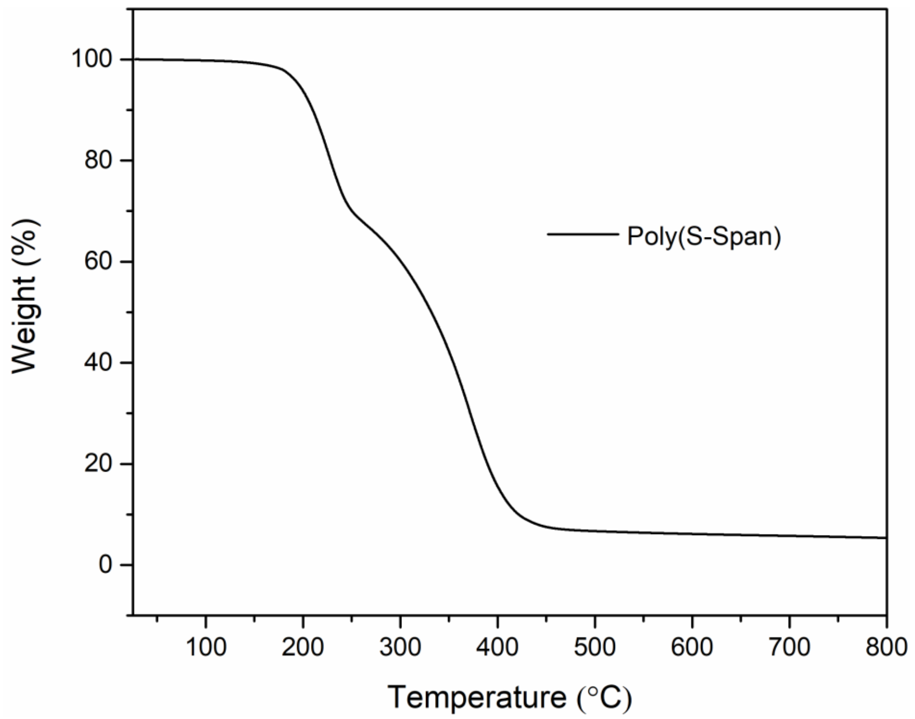

Figure S9. TGA curve of linear polymer poly (S-Span). As shown in figure, the linear polymer shows a good thermal stability and the $T_{\text{deg,5\%}}$ (ºC) of the polymer is 190 °C.

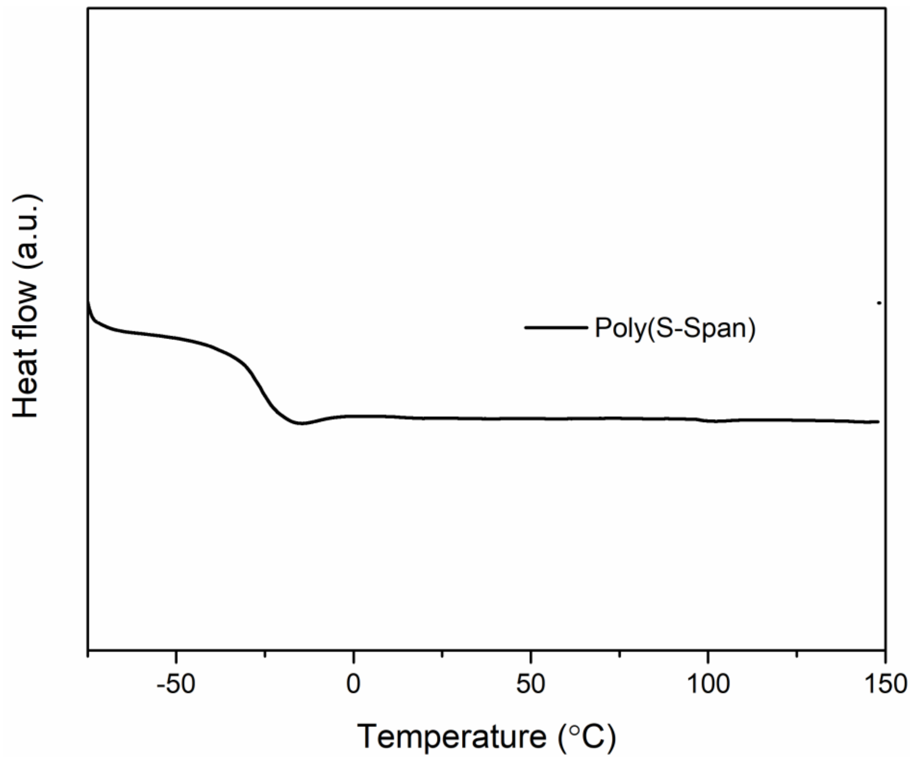

Figure S10. DSC curve of linear polymer poly (S-Span). From DSC, there is no crystalline sulfur melting peak at ~110 °C appeared, suggesting sulfur was fully consumed. A clear T_g of -26.2 °C for this linear polymer was obtained.

Synthesis and characterization of crosslinked polymer S-Span-MDI-X

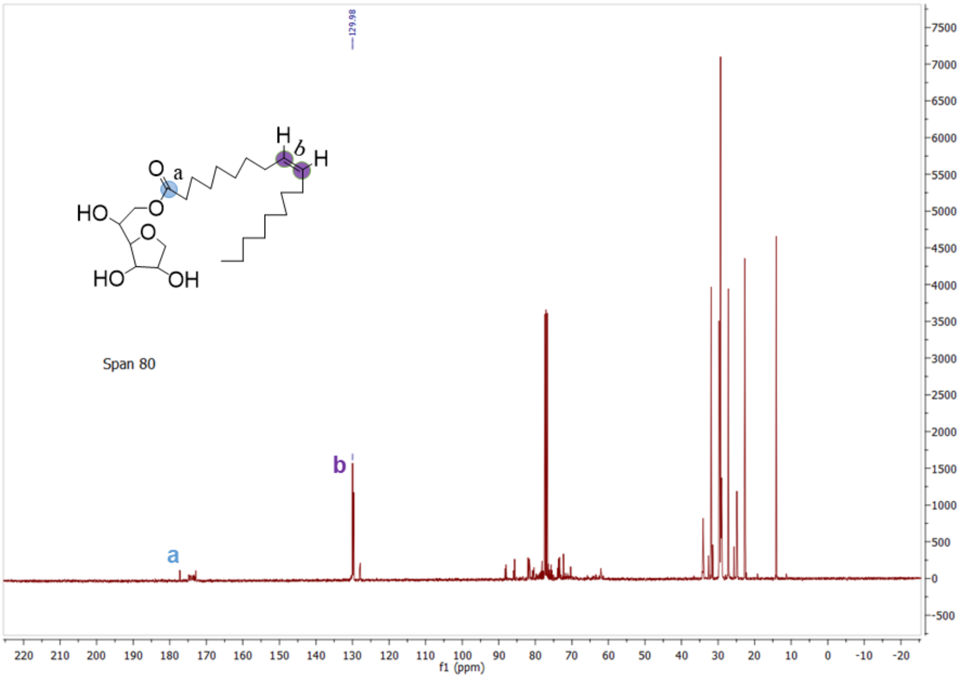

Figure S11. Solution ^{13}C NMR spectrum of pure monomer Span in $CDCl_3$. Different colours were used to mark different carbons. Two peaks marked (at ~130 ppm and ~175 ppm) are responding to C=C and C=O bonds, respectively.

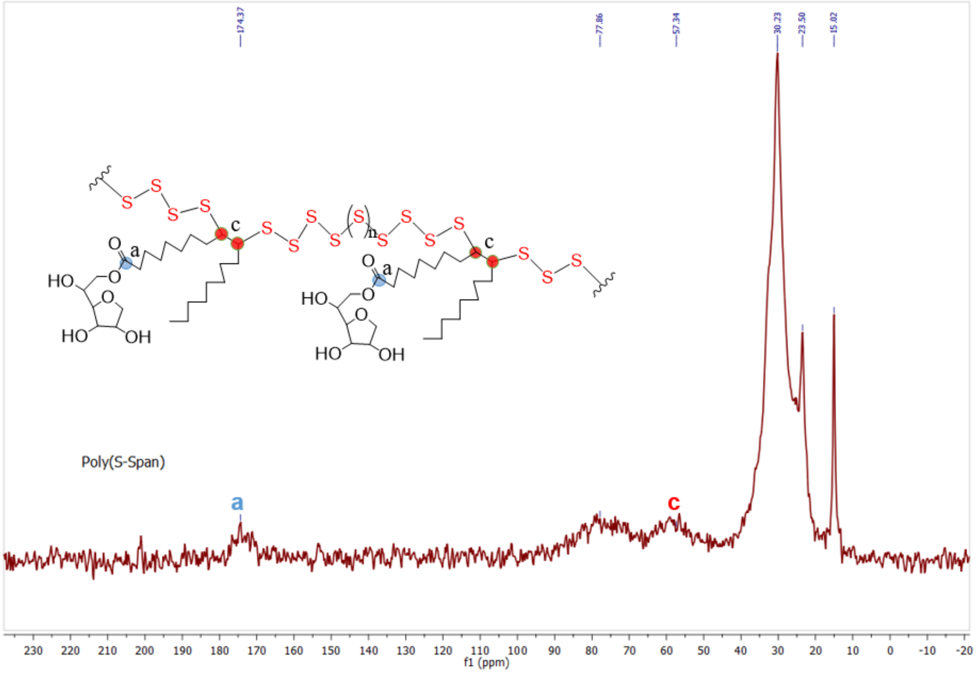

Figure S12. Solid ^{13}C NMR spectrum of linear polymer poly (S-Span). Compared to the spectrum of Span, the peak belonging to C=C have totally disappeared and a new peak (~57 ppm) attributed to C-S appeared. That suggests double carbon bonds in monomer Span were fully consumed by sulfur chains, and a sulfur-based linear polymer was obtained.

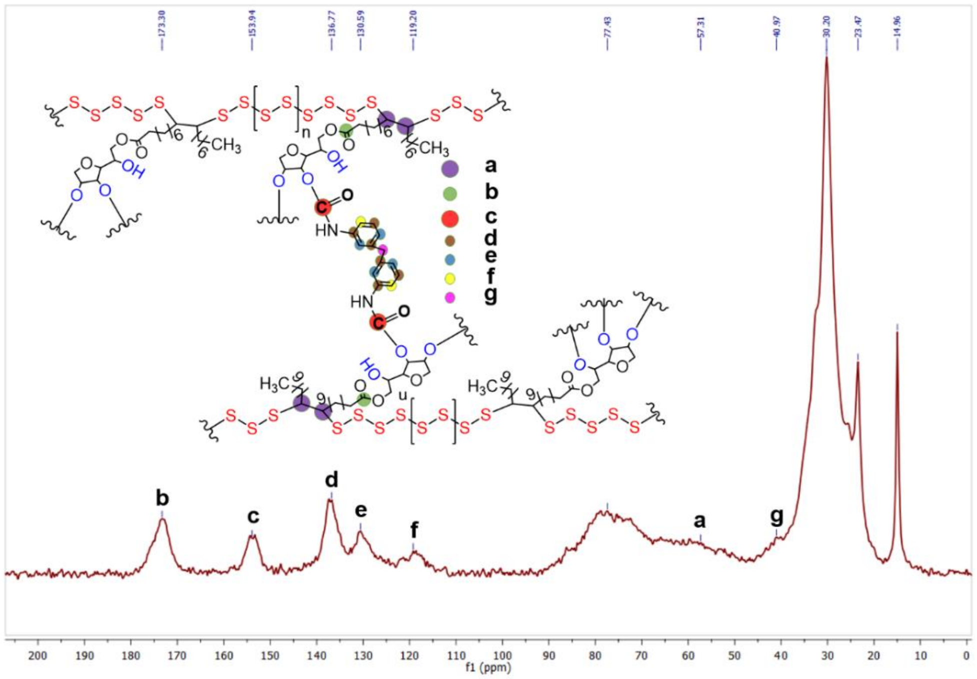

Figure S13. Solid ^{13}C NMR spectrum of crosslinked polymer S-Span-MDI-4. Different colours were used to mark different carbons. New bond C-S responding to peak (a) at ~57 ppm was formed, indicating the polymer network maintains the backbone of sulfur chains. And compared to the ^{13}C NMR spectrum of poly (S-Span), new peak (c) at ~154 ppm was formed, which was attributed to the chemical shift of carbon (red colour carbon) in -NHCOO- bond. It indicates that crosslinked sulfur-base polymer was formed through crosslinker MDI reacting with -OH group in side chains of linear polymer poly (S-Span).

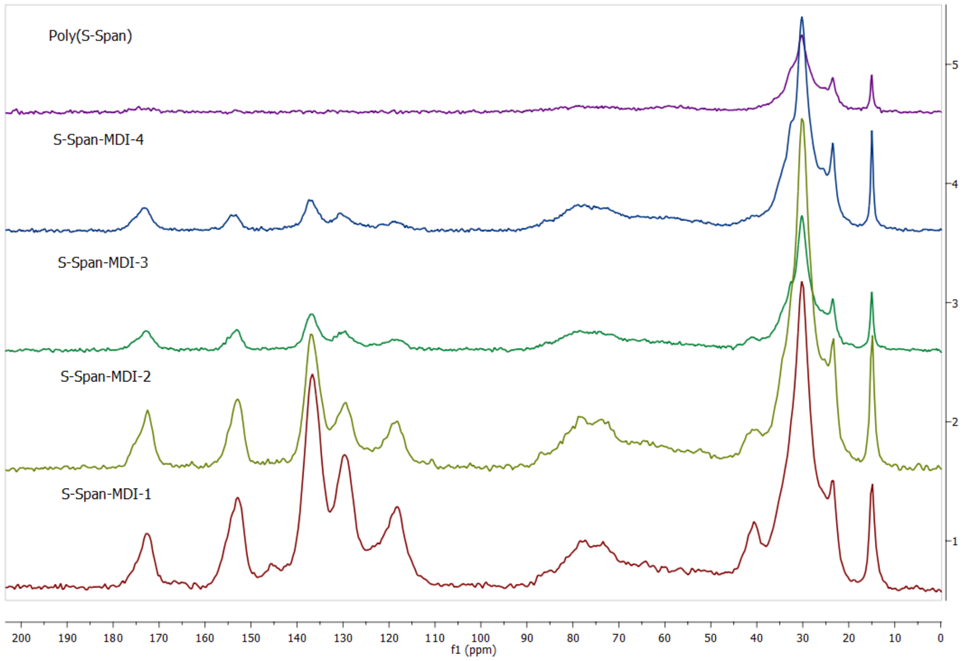

Figure S14. Solid ^{13}C NMR spectra of crosslinked polymers. It is clear that same results with S-Span-MDI-4 were obtained for other three crosslinked polymers. And figure shows that the percentage of MDI was increasing with the increase of theoretical crosslinking degree as designed.

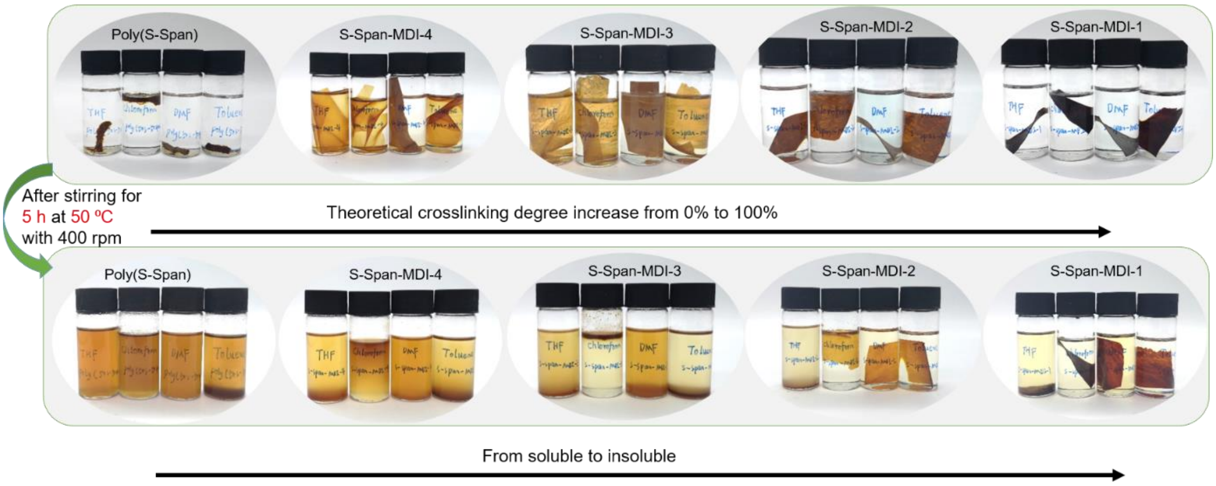

Figure S15. Solubility results. From figure, we can see that as the theoretical crosslinking degree increases, the solubility of polymers in the solvents generally decrease. After stirring for 5 h at 50 °C, the linear polymer was completely dissolved into THF, Chloroform and DMF form a transparent solution, but crosslinked polymers showed a clear insoluble performance tendency varying from partially soluble suspension to totally insoluble swelling solid in four solvents, even through in the best solvent THF for sulfur-polymer, as the increase of theoretical crosslinking degree.

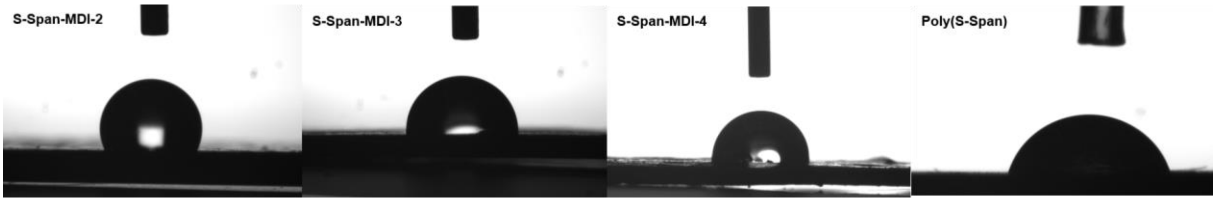

Figure S16. Contact angles of polymers with water. The fitted angles were 113.66° ± 2.59°, 97.32°±6.17°, 91.74°±1.48° and 77.04°±4.35°, responding to polymer S-Span-MDI-2, S-Span-MDI-3, S-Span-MDI-4 and Poly (S-Span), respectively.

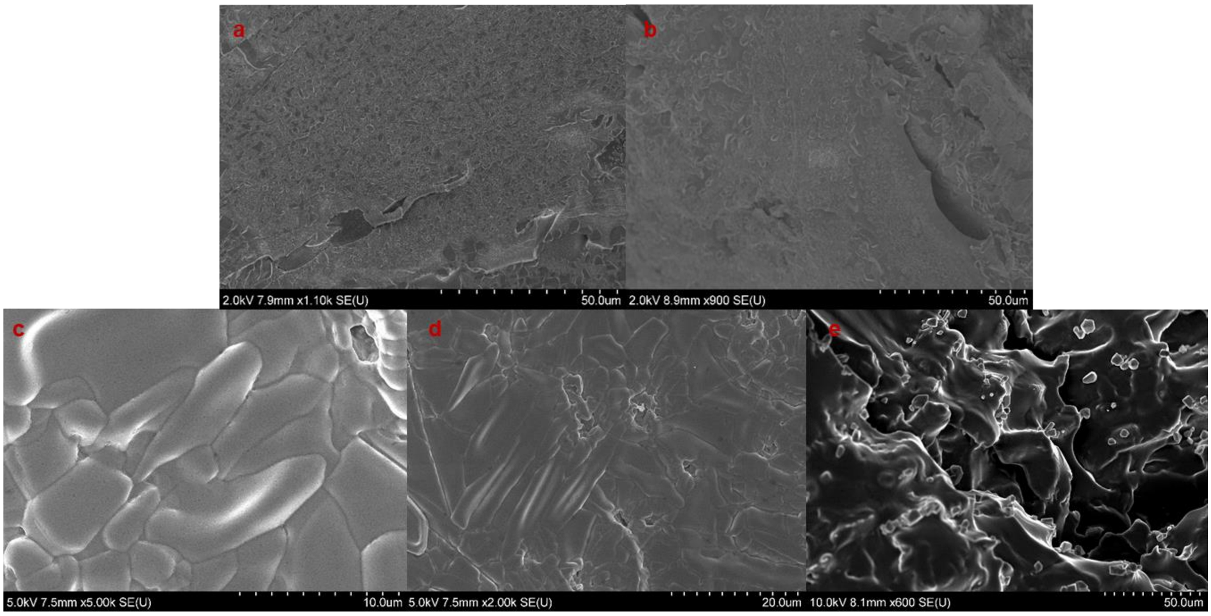

Figure S17. SEM images of fracture topography after tensile test for polymer (a) S-Span-MDI-1, (b) S-Span-MDI-2, (c) S-Span-MDI-3, (d) S-Span-MDI-4 and (e) Poly (S-Span).

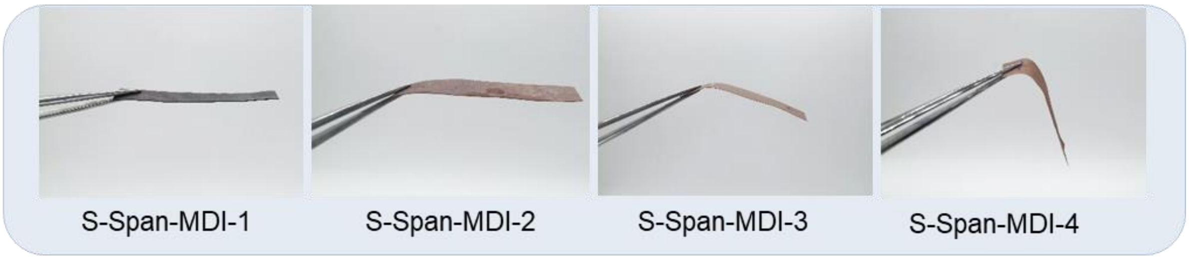

Figure S18. Photographs of crosslinked polymers to show their hardness. Four crosslinked polymers were controlled at same thickness then were pinched by tweezer. It is clear that S-Span-MDI-4 naturally hangs down, which is most soft, and S-Span-MDI-1 remained rigid without shape change, which is most hard. And there is an obvious trend of rigidity with the increase of theoretical crosslinking degree.

Characterization of reprocessed crosslinked polymer S-Span-MDI-X

The crosslinked polymers S-Span-MDI-X were reprocessed using a hot press. The detailed experimental procedure has been discussed in the previous section. The first reprocessed samples were named as R1-S-Span-MDI-X and the second reprocessed samples were named as R2-S-Span-MDI-X. Characterisations of those polymers are shown below.

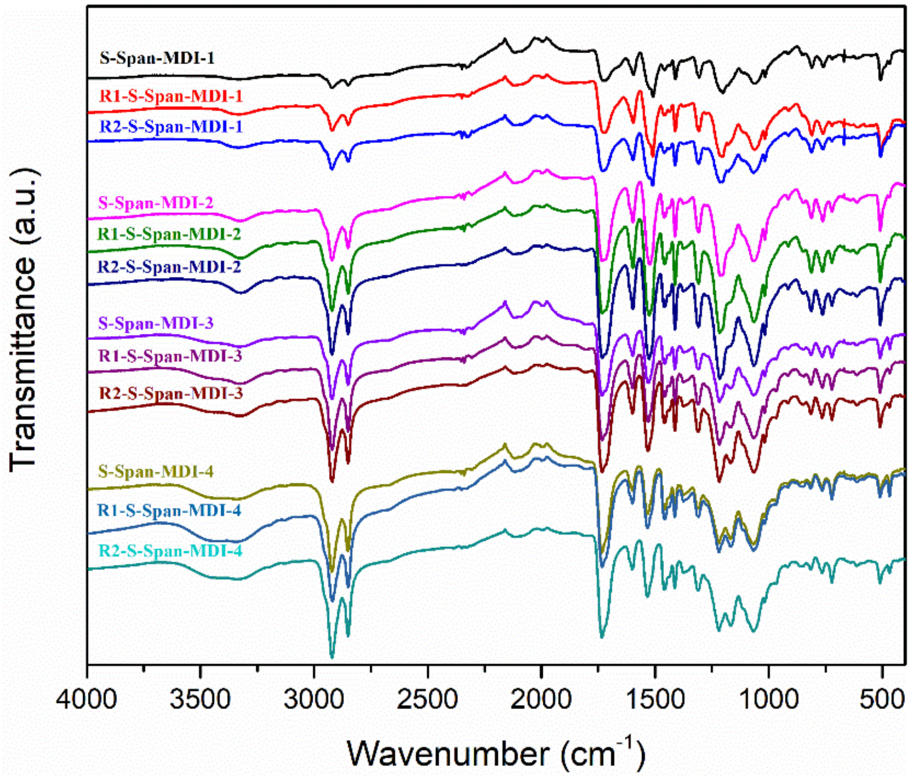

Figure S19. FT-IR traces of reprocessed samples. There is no structure change after reprocessing can be seen from figure.

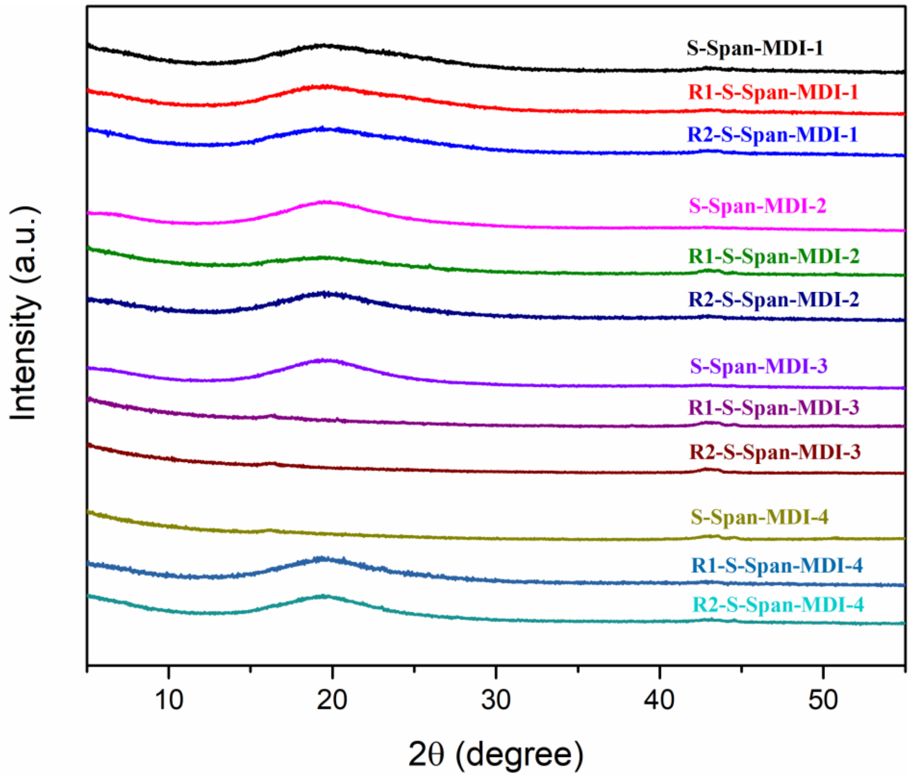

Figure S20. Offset PXRD traces of reprocessed samples. From figure, we can see that after reprocessing, there is little sulfur precipitated out as small crystal signals were observed. That caused by back bite of sulfur chains during the dynamic exchange reaction. Whereas, this has a little effect on the property of polymer and it can be ignored.

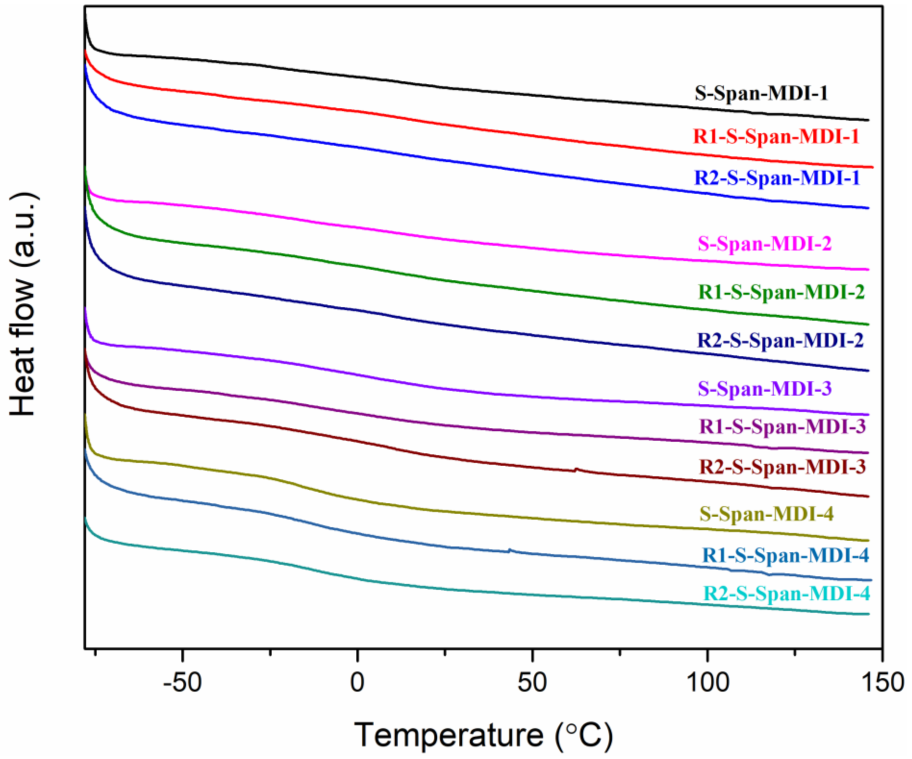

Figure S21. DSC traces of reprocessed samples. As shown in figure, it can be seen that the T_g of polymers slightly decreased after reprocessing, resulting higher strain of reprocessed samples under stretching force.

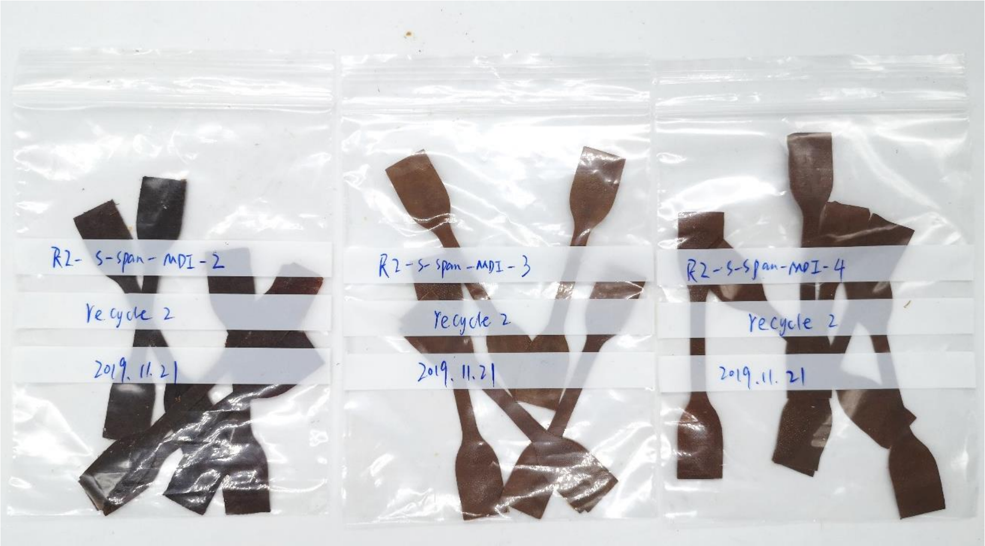

Figure S22. Photographs of second reprocessed dog-bone samples, as used for tensile testing.

References

[1] Delahaye, M.; Winne, J. M.; Du Prez, F. E., Internal Catalysis in Covalent Adaptable Networks: Phthalate Monoester Transesterification As a Versatile Dynamic Cross-Linking Chemistry. *J Am Chem Soc* **2019,** *141* (38), 15277-15287.
